# Epidemiological and Virological Characteristics of Influenza in Chongqing, China, 2011-2015

**DOI:** 10.1371/journal.pone.0167866

**Published:** 2016-12-09

**Authors:** Li Qi, Yu Xiong, Bangzhong Xiao, Wenge Tang, Hua Ling, Jiang Long, Dayong Xiao, Han Zhao, Sheng Ye, Shuang Chen, Zhen Yu, Qin Li

**Affiliations:** Chongqing Municipal Center for Disease Control and Prevention, Chongqing, China; AOU Città della Salute e della Scienza di Torino, ITALY

## Abstract

**Background:**

Chongqing is the largest municipality and located in Southwestern of China, with over 30 million registered inhabitants. There are few reports regarding the epidemiology of influenza in Chongqing. The objective of the paper is to explore the epidemiology of influenza in Chongqing, in order to provide scientific basis for prevention and control of influenza.

**Methodology /Principal Findings:**

From 2011 to 2015, we collected information on influenza-like illness (ILI) patients fulfilling the case definition, and took nasalpharyngeal or throat swabs specimens from ILI cases per week at the 7 sentinel hospitals. Specimens were tested by reverse transcription-polymerase chain reaction(RT-PCR) for influenza. Descriptive epidemiology was applied to analyze the epidemiology and etiology of influenza. A total of 9,696,212 cases were enrolled, of which 111,589 were ILI. Of those 24,868 samples from ILI cases, 13.3% (3,314/24,868) tested positive for influenza virus (65.7% influenza A, 34.1% influenza B, and 0.2% influenza A and B co-infection). Among the influenza A viruses, 71.3% were seasonal influenza A(H3N2) and 28.7% were influenza A(H1N1)pdm09. No cases of seasonal A(H1N1) were detected. The isolation rate was highest in children aged 5–14 years old. Influenza activity consistently peaked during January-March in 2011–2015, and June-July in 2012, 2014 and 2015.

**Conclusions:**

Influenza is an important public health problem among ILI patients in Chongqing, especially among school-aged children. It might be beneficial to prioritize influenza vaccination for school-aged children and implement the school-based intervention to prevent and mitigating influenza outbreaks in Chongqing, particularly during the seasonal peaks.

## Introduction

Influenza is a common vaccine-preventable disease and a major cause of mortality and morbidity globally. According to World Health Organization (WHO), it is estimated that about 5%– 10% of adults and 20%– 30% of children globally are affected by influenza annually, resulting in about 3 to 5 million cases of severe illness, and about 250000 to 500000 deaths [1]. Recently, novel influenza strains have continued to emerge, and China is considered to be an epicenter for influenza new strains, such as type A/H2N2 in the 1950s, A/H3N2 in the 1960s, and A/H5N1 occurred in Hong Kong in 1997.

Chongqing is the largest municipality, with over 30 million registered inhabitants, and is located in the subtropical region at latitude 29.6°N in Southwestern of China. Influenza surveillance was initiated in 2005 in Chongqing, including 4 sentinel hospitals. There are few reports regarding the epidemiology of influenza in Chongqing, which in turn hampered identifying the optimal region-specific policy to diminish disease burden. The previous study has shown the seasonality pattern of influenza in China from 2005 to 2010 [2], and documented no marked seasonal patterns was observed in Chongqing by stratified analysis at a regional level, partly due to the relatively few influenza virus data sets. To better understand the epidemiological and virologic characteristics of influenza in Chongqing, the surveillance was extended to 3 additional sentinel hospitals since 2011.

So, we summarize the findings of expanded influenza surveillance in Chongqing during 2011–2015, when the surveillance was fully implemented, and provide a scientific basis for prevention and control of influenza.

## Materials and Methods

### Sentinel hospitals

Influenza surveillance was conducted in 7 sentinel hospitals in Chongqing from 2011 to 2015. The 7 sentinel hospitals were selected based on higher accessibility to patients, higher qualifications of medical staff, adequate specimen storage capacity, and the desire of the physicians and nurses to participate voluntarily in the surveillance program. Sentinel hospitals included four rural comprehensive medical institutions (Qianjiang Central Hospital, Wanzhou Central Hospital, Fulin Central Hospital and Yongchuan Hospital of Chongqing Medical University), two urban comprehensive medical institutions (Banan Central Hospital and the First Affiliated Hospital of Chongqing Medical University) and one urban pediatric hospital (Children’s Hospital of Chongqing Medical University).

At each sentinel hospital, physicians or nurses were trained in surveillance methods, including influenza-like illness (ILI) cases identification, obtaining consent, specimen collection, storage, and transportation.

### Influenza-like illness surveillance

At each sentinel hospital, trained nurses and clinicians collected data on the counts of visits and the total number of ILI to outpatient and/or emergency departments, using the World Health Organization case definition. ILI case was defined as a person with fever (≥38.0°C), cough and/or sore throat, and absence of other diagnoses [3, 4]. The number of total visits and ILI cases was reported by age groups (0–4, 5–14, 15–24, 25–59, ≥ 60) to a centralized online system maintained by Chinese Center for Disease Control and Prevention.

The percentage of visits for ILI was defined as the number of ILI cases among the total number of visits, expressed as a percentage (ILI%), and calculated for each calendar week of this study.

### Influenza virological surveillance

The enrolment criteria for influenza virological surveillance included ILI cases in sentinel hospitals and no treatment with antiviral medication. Nasopharyngeal swabs or throat swabs specimens were collected and immediately immersed into a sterile tube, containing virus transport medium, kept refrigerated at each sentinel sites at 4°C (no longer than 48 hours). A quota of 10–20 specimens was targeted from each sentinel hospital and transported once a week (Friday) to the territorial Center for Disease Control and Prevention (CDC) laboratory, where specimens were stored at -70°C until tested for influenza viruses.

Specimens were analyzed for influenza A or B, and those positive for influenza A were further confirmed to determine seasonal H1N1, H3N2 and H1N1(pdm09) by reverse transcription-polymerase chain reaction (RT-PCR) using primers, probes and reagents recommended by Chinese CDC in territorial CDC laboratory, following a standard protocol Chinese Center for Disease Control and Prevention (2010) [5]. Test results for the RT-PCR were to be returned to the treating physicians within one week of specimen delivery. Meanwhile, participating physicians completed sample information sheets, including the demographic information, chief complaint, and data of ILI onset and hospital visit. The related information about the patients was input into the Chinese influenza Surveillance Net.

**Fig 1:**
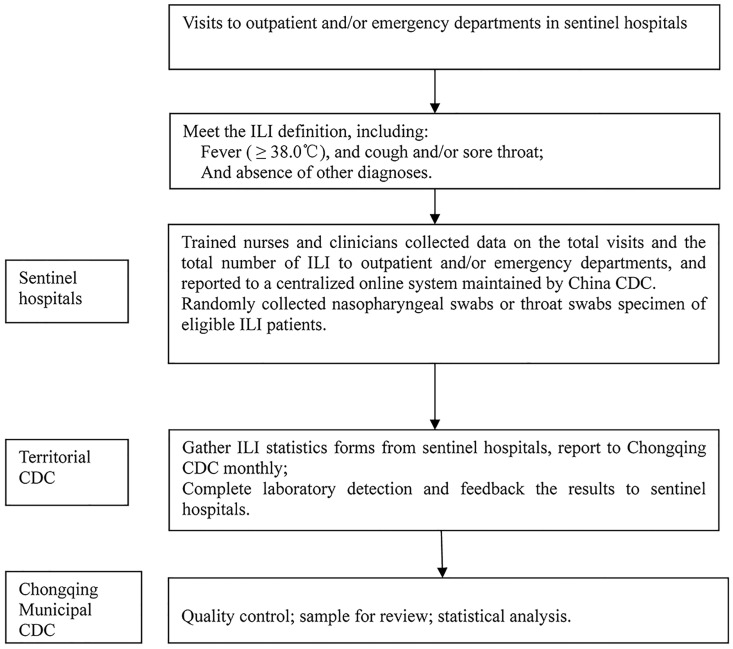
Influenza surveillance flowchart. Abbreviation: ILI, influenza-like-illness; CDC, center for disease control and prevention.

Below are the data and specimen collection procedure (Fig 1).

### Ethical considerations

The protocol was approved by the department of China Health and Family Planning Commission (previously called China Ministry of Health) as part of the monitoring of disease with epidemic potential and therefore did not require ethical approval. Verbal and written informed consent were obtained from patients who were ≥ 18 years, and proxy consent was obtained from their legal guardians for participant < 18 years.

### Statistical analysis

Data management and statistical analysis were performed using Statistical Package for Social Sciences 18.0. Histogram and line charts were used to show the time distribution of ILI cases and positive influenza. The Chi-square test was conducted to test differences in categorical variables. A two tailed p value < 0.05 was considered statistically significant.

## Results

### Epidemiological characteristics of ILI

During the 5-year surveillance, a total of 9,696,212 patients visits were recorded in the 7 sentinel hospitals. Of these, 111,589 (1.2%) were patient visits for ILI. Children aged under 5-year olds constituted 55.3% of the total number of ILI cases, followed by the 5–14 years old age group, which accounted for 28.0% of ILI cases. Individuals aged 60 years and older accounted for 2.7% of the ILI cases, whose number of the cases was the least.

**Fig 2:**
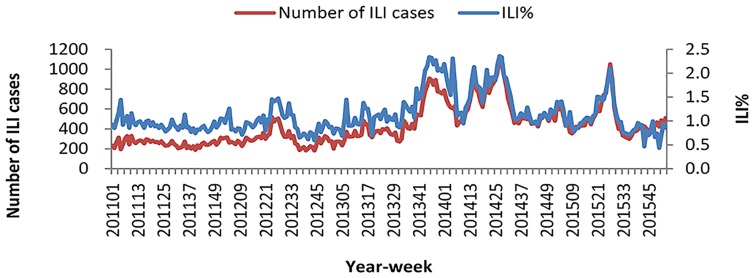
The weekly distribution of influenza-like illness, 2011–2015.

The weekly number of total ILI consultations and ILI% were shown in Fig 2. ILI cases were reported throughout the year and did not show any clear seasonal pattern.

### Etiological characteristics

Overall, 24,868 specimens from ILI cases were collected for virus detection in 2011–2015. Among tested specimens, 13.3% (3,314/24,868) were positive for influenza virus by RT-PCR (Table 1).

**Table 1:** The frequency of influenza viruses and proportion for influenza type /subtype by year and demographic characteristics.

Characteristics	No. Samples tested	No.(frequency) of influenza cases	No.(proportion) of influenza cases			
			Seasonal A(H3N2)	A(H1N1) pdm09	Type B	Type A&B
**Year**						
2011	3237	366(11.3)	26(7.1)	158(43.2)	182(49.7)	0
2012	3781	754(19.9)	467(61.9)	4(0.5)	283(37.5)	0
2013	5962	752(12.6)	91(12.1)	504(67.0)	154(20.5)	3(0.4)
2014	5640	703(12.5)	446(63.4)	30(4.3)	226(32.2)	1(0.1)
2015	6221	739(11.9)	453(61.3)	0	285(38.6)	1(0.1)
**Gender**						
Male	13757	1879(13.7)	850(45.2)	406(21.6)	620(33.0)	3(0.2)
Female	11084	1435(12.9)	633(44.1)	290(20.2)	510(35.5)	2(0.1)
**Age group**						
0 to 4 years	9880	876(8.9)	381(43.5)	224(25.6)	270(30.8)	1(0.1)
5 to 14 years	7421	1354(18.2)	476(35.2)	231(17.1)	645(47.6)	2(0.1)
15 to 24 years	2126	322(15.1)	180(55.9)	69(21.4)	73(22.7)	0
25 to 59 years	4296	621(14.5)	356(57.3)	153(24.6)	110(17.7)	2(0.3)
Over 60 years	1118	141(12.6)	90(63.8)	19(13.5)	32(22.7)	0
**Total**	24841	3314(13.3)	1483(44.7)	696(21.0)	1130(34.1)	5(0.2)

The median age of influenza cases was 8 years old (range: 1 month to 91 years). Isolation rates showed significant variation on the basis of age (X^2^ = 3.366, *p* = 0.000), the highest isolation rate was seen in children aged 5–14 years (18.2%), followed by persons aged 15–24 years (15.1%) and 25–59 years (14.5%), the lowest rate was seen in children aged less than 5 years (8.9%). There was no observed difference in the proportion of specimens positive for influenza by gender (13.7% in males and 12.9% in females) (Table 1).

**Fig 3:**
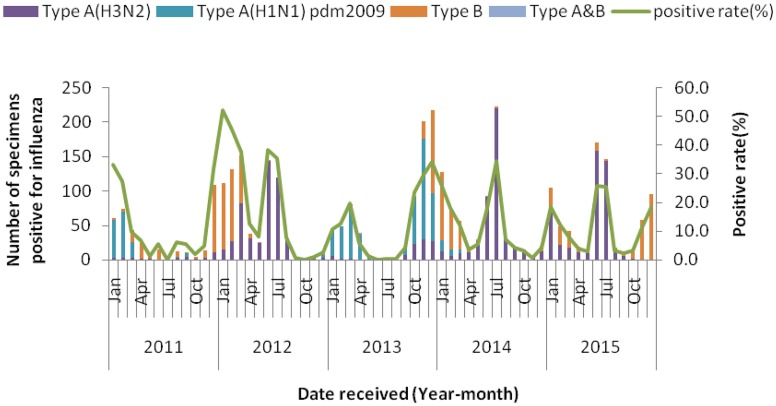
Monthly distribution of influenza isolates from 2011 to 2015.

The isolation rates showed significant variation during the 5 surveillance years (X^2^ = 1.721, *p* = 0.000), varied from 11.3% in 2013 to 19.9% in 2012. In contrast to the reported ILI cases, we observed seasonal peaks in positive influenza cases (Fig 3). In 2011, the highest isolation rate occurred in January-March. In 2012–2015, a bio-modal seasonal pattern was observed, with two isolation rates peaks occurred in January-March and June-July in 2012, 2014 and 2015, and occurred in January-March and October-December in 2013. During June-July peaks were found mostly dominated by seasonal A(H3N2) (Fig 3).

Of the 3314 cases tested positive for influenza virus, 2079 cases were influenza A positive, 1130 cases were influenza B, and the other 5 cases were influenza A and B co-infection, accounting for 65.7%, 34.1% and 0.2% of total isolates, respectively. After sub-typing the influenza A viruses, 71.3% (1483/2079) cases were seasonal A(H3N2), 28.7% (596/2079) cases were influenza A(H1N1) pdm09. No cases of seasonal influenza A(H1N1) were detected (Table 1).

According to the bar charts showing influenza subtypes (Fig 3), the advantage influenza strain changed by year. In 2011, influenza A(H1N1) pdm09 and influenza B virus combined to account for the vast majority of isolates (43.2% and 49.7%). In 2012, 2014 and 2015, seasonal A(H3N2) was the dominant subtype, accounting for 61.9%, 63.4% and 61.3% of isolates, respectively. In 2013, influenza A(H1N1) pdm09 viruses accounted for 67.0% and became the dominant circulating subtype.

**Fig 4:**
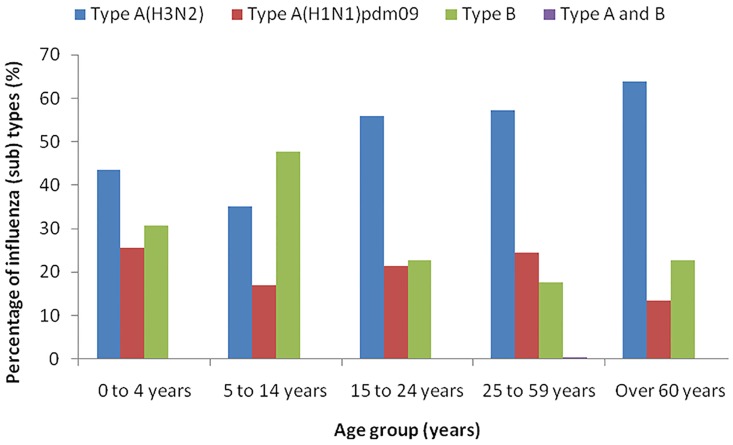
Age distribution of influenza virus types and subtypes.

Fig 4 presents the age distribution of influenza virus types and subtypes. In influenza cases aged 5–14 years, 47.6% tested positive for influenza B virus, whereas influenza A was predominant influenza type among other age groups (Table 1 & Fig 4). Of the influenza A virus subtypes, seasonal A (H3N2) virus dominated over A(H1N1) pdm09 in all age group cases.

## Discussion

This study is the first to describe the epidemiology and virological of influenza in Chongqing from 2011 to 2015. Data from sentinel surveillance of influenza showed that influenza virus is an important cause of ILI in Chongqing, especially among school-age children.

Although influenza viruses circulate throughout the year, the proportion of ILI patients with influenza varied from year-to-year and showed two possible peaks, one in January-March in the spring season, another might be in June-July in the summer season (expect for 2011 and 2013). The observed influenza seasonality was similar to other regions at the same latitude (latitude 27.4°N -31.3°N) in China [2]. Several factors have been proposed to explain the seasonality of influenza, including climate conditions (temperature and humidity), living environment, host susceptibility and virus characteristics etc. Influenza virus survival and transmissibility might be influenced by multiple factors. Previous studies have found the seasonality of influenza viruses was associated with rainy season [6–12], or with low humidity levels [13–15]. Therefore, additional years of surveillance data with more information on climatic factors might be helpful to better understand the seasonality of influenza in Chongqing.

Although all age groups were affected with influenza, as had been observed elsewhere, school-aged children, 5–14 years old were more frequently infected [10, 16–19], with the highest percentage of influenza virus infections among all age groups, while children aged < 5 years had the lowest isolation rate. However, ILI cases were highest among children aged < 5 years old. This phenomenon could be explained by the fact that besides influenza virus, other pathogens, such as rhinoviruses, respiratory syncytial viruses (RSV), and enteroviruses, might be more likely to infect small children aged < 5 years [9, 20–22]. Thus, more respiratory viral etiologies should be tested in the surveillance system in future, especially among children < 5 years. In our study, influenza B was common among children aged 5–14 years old, previous studies showed that influenza B virus led to a substantial morbidity and mortality burden [23–26]. Given the higher proportion of influenza among school-based children, it might be beneficial to prioritize influenza vaccination for school-aged children (5–14 years old) and implement school-based intervention (eg. hand-washing program) to prevent and mitigating influenza outbreaks in Chongqing [19, 27].

The virological data from the sentinel influenza surveillance system revealed that multiple influenza virus subtypes co-circulated in Chongqing and the predominant subtype varied each year. In total, seasonal influenza A(H3N2) was the advantage strains in Chongqing and accounted for 44.7% of positive specimens, which was the main influenza strains in 3 of 5 years from 2011 to 2015, consistent with finding from other studies [17, 18, 28, 29]. Influenza A(H1N1) pdm09 was the predominant virus type in 2011 and 2013, two and four years after the 2009 pandemic outbreak, which was supported by a statement released by World Health Organization in 2010, which says that the pandemic strains may circulate as seasonal viruses for few more years, causing occasional outbreaks [30]. It is interesting to note that low virus isolation in June-July was observed in 2011 and 2013 when influenza A(H1N1)pdm09 was the predominant subtype. This indicated that influenza A(H1N1)pdm09 may lead to a single annual peak in Chongqing, which needed to be explored by many more years of surveillance data. Furthermore, through June-July peaks in 2012, 2014 and 2015 were mostly caused by seasonal A(H3N2), indicating that seasonal epidemic of A(H3N2) virus might be transmitted quiet easily during summer seasons. There were no cases of influenza H1N1 detected in 2015, which might be explained by the fact that our surveillance work relied upon a convenience sample of ILI cases in 7 sentinel hospitals. It might possible that some of H1N1 virus infections were missed in the data. Therefore, it is crucial to expanding sentinel sites in more regions for better understanding the epidemiology and virological characteristics of influenza in Chongqing.

While the information provided by this study is valuable, there may be several limitations. First, our surveillance data focused only on patients with ILI from 7 sentinel hospitals. So the result may not be representative of the entire population of Chongqing. Second, we did not detect non-influenza respiratory pathogens in this study, such as RSV, adenovirus, and rhinovirus, which hampered understanding the spectrum of ILI cases. Finally, while we were able to obtain epidemiologic and virological data of influenza, the burden of influenza cannot be estimated by the influenza surveillance system.

## Conclusions

In summary, our findings demonstrated that influenza is an important public health problem among ILI patients in Chongqing, especially among school-aged children. Influenza viruses circulate throughout the year, with two possible peaks, one in January-March, another might be in June-July, prevention and control strategies should be focused upon the seasonal peaks. Multiple influenza virus subtypes co-circulated and the predominant subtype varied each year. In addition to strengthening epidemiological and virological data collection of influenza, collecting more information on meteorological factors, environment data and economic impact of influenza is warranted. These additional data can provide a better understanding of the epidemiology and impact of influenza, which is vital for making optimal region-specific policy to diminish disease burden in Chongqing.

## Supporting Information

S1 File Supporting file. (XLSX)
